# Macrophages and Their Role in Atherosclerosis: Pathophysiology and Transcriptome Analysis

**DOI:** 10.1155/2016/9582430

**Published:** 2016-07-17

**Authors:** Yuri V. Bobryshev, Ekaterina A. Ivanova, Dimitry A. Chistiakov, Nikita G. Nikiforov, Alexander N. Orekhov

**Affiliations:** ^1^Institute of General Pathology and Pathophysiology, Russian Academy of Medical Sciences, Moscow 125315, Russia; ^2^Faculty of Medicine, School of Medical Sciences, University of New South Wales, Kensington, Sydney, NSW 2052, Australia; ^3^School of Medicine, University of Western Sydney, Campbelltown, NSW 2560, Australia; ^4^Department of Development and Regeneration, KU Leuven, 3000 Leuven, Belgium; ^5^Department of Molecular Genetic Diagnostics and Cell Biology, Institute of Pediatrics, Research Center for Children's Health, Moscow 119991, Russia; ^6^Institute for Atherosclerosis, Skolkovo Innovation Center, Moscow 143025, Russia; ^7^Department of Biophysics, Biological Faculty, Moscow State University, Moscow 119991, Russia

## Abstract

Atherosclerosis can be regarded as a chronic inflammatory state, in which macrophages play different and important roles. Phagocytic proinflammatory cells populate growing atherosclerotic lesions, where they actively participate in cholesterol accumulation. Moreover, macrophages promote formation of complicated and unstable plaques by maintaining proinflammatory microenvironment. At the same time, anti-inflammatory macrophages contribute to tissue repair and remodelling and plaque stabilization. Macrophages therefore represent attractive targets for development of antiatherosclerotic therapy, which can aim to reduce monocyte recruitment to the lesion site, inhibit proinflammatory macrophages, or stimulate anti-inflammatory responses and cholesterol efflux. More studies are needed, however, to create a comprehensive classification of different macrophage phenotypes and to define their roles in the pathogenesis of atherosclerosis. In this review, we provide an overview of the current knowledge on macrophage diversity, activation, and plasticity in atherosclerosis and describe macrophage-based cellular tests for evaluation of potential antiatherosclerotic substances.

## 1. Introduction

**Figure 1:**
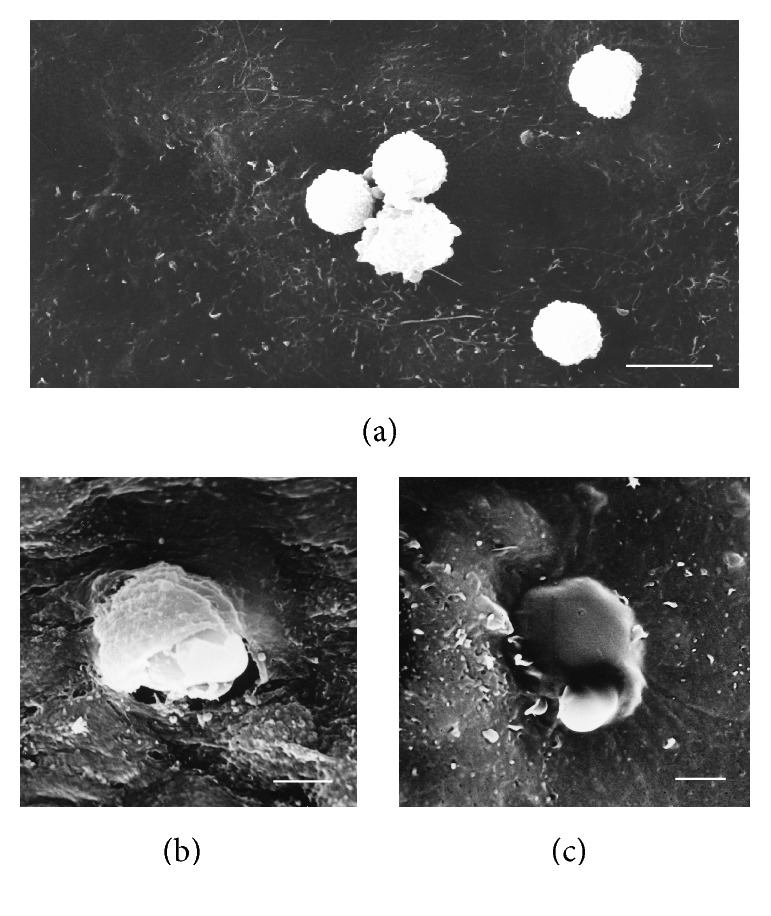
Adhesion (a) and penetration (b, c) of blood monocytes into the intima of the human aorta. Scanning Electron Microscopy (SEM). Scale bars = 15 *μ*m (a) and 5 *μ*m (b, c).

Atherosclerosis is a chronic inflammatory disease triggered by lipid retention in the arterial wall [1]. Certain areas of arteries, such as branching points and bends, are especially prone to atherosclerotic lesion development due to local disturbance of endothelial function. In such areas, circulating lipoprotein particles can penetrate into the arterial wall and accumulate in the subendothelial proteoglycan-rich layer of the arterial wall intima. According to current understanding, low-density lipoprotein (LDL), especially in its modified form, serves as a primary source of lipid accumulation in atherosclerotic lesions [2]. Atherogenic modification of LDL includes oxidation, enzymatic processing, desialylation, and aggregation. These modifications render the lipoprotein particles proinflammatory and induce an immune response leading to the formation of circulating LDL-containing immune complexes that are highly atherogenic [3]. Macrophages play a decisive role at all stages of atherosclerotic lesion progression [4, 5]. It is widely accepted that circulating monocyte-derived cells are recruited to the atherosclerotic lesion site (Figure 1), where they differentiate into macrophages. A number of recent studies, however, challenged this paradigm by demonstrating that most tissue macrophages develop independently of monocyte input from precursor cells present in adult tissues [6]. Interestingly, subendothelial intimal layer of human arterial wall contains a population of pluripotent pericyte-like cells that can differentiate into various cell types including phagocytes, positive for macrophage marker CD68 [7]. Macrophages in atherosclerotic lesions actively participate in lipoprotein ingestion and accumulation giving rise to foam cells filled with lipid droplets. Accumulation of foam cells contributes to lipid storage and atherosclerotic plaque growth. Macrophages populating the atherosclerotic plaque have a decreased ability to migrate, which leads to failure of inflammation resolution and to further progression of the lesion into complicated atherosclerotic plaque [8]. At this stage, macrophages contribute to the maintenance of the local inflammatory response by secreting proinflammatory cytokines and chemokines and producing reactive oxygen species. Dying macrophages are responsible for necrotic core formation in progressing plaques [9]. The key role that macrophages play in the pathogenesis of atherosclerosis makes them an attractive target for therapy development. Several possibilities have been considered, including inhibiting monocyte/macrophage recruitment to growing lesions, stimulating cholesterol efflux and diminishing lipid storage, and taking advantage of macrophage plasticity and the ability to polarize towards pro- or anti-inflammatory phenotypes [5].

## 2. Mononuclear Phagocyte System and the Role of Macrophages

According to the classical view, monocytes and macrophages form a continuous system, the mononuclear phagocyte system, which plays a central role in the innate immune response [10]. Circulating monocytes are recruited to the sites of injury or pathogen invasion by specific signals, including cytokines and chemokines released by tissue cells. At the lesion site, monocytes differentiate to macrophages that actively take part in the immune response by engulfing pathogens and damaged cells via phagocytosis and releasing proinflammatory factors. On the other hand, macrophages are also responsible for the resolution of the inflammatory response and tissue remodelling. The classical system regarded macrophages as terminally differentiated cells that are constantly renewed by monocytes newly recruited from circulation. This understanding was based primarily on tracing radiolabelled differentiating monocytes/macrophages in mice during inflammatory response. However, more recent studies have demonstrated that the ontogeny of tissue macrophages is more complex, and a large proportion of these cells derives from resident precursors [6].

Studying of monocyte/macrophage heterogeneity is challenging, because different subpopulations of these cells defined by expression of specific markers do not completely overlap in mice and humans. In both organisms, circulating monocytes can be divided into several distinct types based on the expression of surface molecules and chemokine receptors [11]. In humans, monocytes positive for CD14 and negative for CD16 surface antigens are the most prevalent and are referred to as classical monocytes [12, 13]. Like murine proinflammatory (LY6C^hi^) monocytes, these cells express CC-chemokine receptor 2 (CCR2). Monocytes positive for CD16 can be further divided into 2 subsets: CD14^+^CD16^++^ (nonclassical) and CD14^++^CD16^+^ (intermediate). Although both subclasses can produce proinflammatory factors, their functions in the organism are different. Nonclassical monocytes have antiviral activity and selectively produce proinflammatory factors in response to viral particles and nucleic acid-containing complexes, patrol the tissues, and are likely to be responsible for the local immune response [14]. Intermediate monocytes are capable of producing large amounts of proinflammatory molecules, such as tumor necrosis factor in response to stimulation [15]. Studying the distinct subpopulations of monocytes and their roles in the pathogenesis of atherosclerosis may help in developing novel therapeutic approaches specifically targeting different stages of the disease progression.

It has been noted that the number of circulating proinflammatory monocytes is significantly increased in animal models of atherosclerosis, such as* Apoe* ^−/−^ mice in comparison to control animals [16]. Hypercholesterolaemia seems to promote the proliferation of haematopoietic stem and progenitor cells and to enhance their sensitivity to granulocyte-macrophage colony-stimulating factor (GM-CSF). On the contrary, production of high-density lipoprotein (HDL), which promotes cholesterol efflux and protects against atherosclerosis, reverses this phenotype [17].

During haematopoiesis, monocyte differentiation into macrophages is triggered mainly by two growth factors: GM-CSF and macrophage colony-stimulating factor (M-SCF) [18]. Differentiation of circulating monocytes can be induced by various stimuli, most importantly, in response to infection or aseptic inflammation. The latter process plays an important role in the pathogenesis of atherosclerosis [19]. Fatty streaks represent the early stages of atherosclerotic lesion development. It has been demonstrated that monocytes can be recruited to fatty streaks and can penetrate into the arterial wall due to the increased endothelial permeability linked to the local endothelial dysfunction. In mice, both proinflammatory and patrolling monocytes can be recruited to growing atherosclerotic lesions by P- and E-selectin-dependent rolling followed by intercellular adhesion molecule 1- (ICAM1-) and vascular cell adhesion molecule 1- (VCAM1-) dependent adhesion [20]. Proinflammatory monocyte migration into the arterial wall is mediated by CCR2, CCR5, and CX_3_C-chemokine receptor 1 (CX_3_CR1) signalling. Correspondingly, inhibition of these molecules in* Apoe* ^−/−^ murine model of atherosclerosis prevented monocyte recruitment and atherosclerotic lesion growth [21]. Chemokines can be produced by activated endothelial cells at the lesion site, as well as by intimal macrophages and resident arterial wall cells. Proinflammatory monocytes penetrating into the arterial wall differentiate into macrophages and contribute to the inflammatory process and lesion development [16]. The role of patrolling monocytes in the disease pathogenesis is less clear. They participate in phagocytosis and might differentiate into dendritic cells [22].

Monocytes differentiating into macrophages demonstrate a number of morphological and structural changes, including enlargement, increase of organelles numbers, intensification of metabolism, enhanced expression of surface receptors, and altered sensitivity to signalling molecules. Differentiating monocytes have increased lysosomal enzyme activity, which prepares them for active phagocytosis and digestion of the engulfed material [23]. Importantly, macrophages that populate atherosclerotic lesions have a decreased ability to migrate. This contributes to the failure of inflammation resolution and to the formation of complicated plaques [5, 24]. In such plaques, different types of immune cells, as well as resident cells of the arterial wall, participate in the inflammatory process by secreting proinflammatory factors and matrix-degrading proteases. The increased cell death leads to the formation of necrotic core in the progressing plaque. On the other hand, recruitment of monocytes to the arterial wall can also be important for inflammation resolution and atherosclerotic lesion regression [25].

## 3. Macrophage Heterogeneity

One of the key features of macrophages is their high degree of plasticity that allows them to produce a fine-tuned response to various microenvironmental stimuli [26, 27]. Such plasticity and heterogeneity made it challenging to achieve a comprehensive macrophage classification. Moreover,* in vitro* studies of macrophage activation and differentiation may not reflect the* in vivo* situation accurately enough, since these processes are fine-tuned by various factors present in the organism's blood and tissues and can be modelled only roughly.

The identification of pro- and anti-inflammatory macrophages led to the establishment of the classical model of macrophage activation. This model defined two main phenotypes of macrophages: proinflammatory M1 and alternative M2. M1 macrophages differentiate in response to toll-like receptor (TLR) and interferon-*γ* signalling and can be induced by the presence of pathogen-associated molecular complexes (PAMPs), lipopolysaccharides, and lipoproteins. These cells secrete proinflammatory factors, such as tumor necrosis factor- (TNF-) *α*, interleukin-1*β* (IL-1*β*), IL-12, and IL-23, and chemokines CXCL9, CXCL10, and CXCL11. Proinflammatory macrophages produce high levels of reactive oxygen species (ROS) and nitric oxide (NO) that also contribute to the development of the inflammatory response (Table 1) [28]. M2 macrophages that have anti-inflammatory properties are induced in response to Th2-type cytokines IL-4 and IL-13 and secrete anti-inflammatory factors, such as IL-1 receptor agonist and IL-10. Macrophages corresponding to M1 and M2 types were described in atherosclerotic lesions. Proinflammatory (M1) macrophages were enriched in progressing plaques, and M2 macrophages were present in regressing plaques, where they were involved in tissue repair and remodelling [28].

Recent studies have demonstrated that the bipolar M1/M2 classification does not accurately describe the macrophage diversity [26]. Therefore, additional classes of macrophages were distinguished depending on activation stimuli. Some authors proposed to divide the M2 type into several subgroups depending on the activation stimuli and protein expression pattern. M2a macrophages induced by IL-4 and IL-13 express high levels of CD206, IL-1 receptor agonist (IL1RN). M2b macrophages can be induced by TLR signalling and immune complexes, as well as IL-1R ligands [27]. They produce both anti-inflammatory (IL-10) and proinflammatory (IL-6, TNF-*α*) cytokines. M2c macrophages that can be induced by IL-10, transforming growth factor-*β* (TGF-*β*), and glucocorticosteroids possess strong anti-inflammatory properties and produce pentraxin-3 (PTX3), TGF-*β*, and IL-10. They express Mer receptor kinase (MERTK) and are responsible for clearance of apoptotic cells [29]. M2d macrophages differentiated in response to TLR signalling through the adenosine A2A receptor were demonstrated to have angiogenic properties that can play a role in tumor progression and atherosclerotic plaque growth [30]. This classification, however, can be further broadened to include species-specific macrophage types. For instance, Mox macrophages were found only in mouse models of atherosclerosis, where they were induced by proatherogenic oxidized LDL. Furthermore, proinflammatory macrophages could be induced by platelet chemokine CXCL4 [31]. They lose the expression of the haemoglobin-haptoglobin scavenger receptor CD163, which is essential for haemoglobin clearance after the plaque haemorrhage and has therefore protective properties in atherosclerosis [32].

The described complexity of macrophage phenotypes urged the development of a more comprehensive classification system to avoid confusion and facilitate interpretation of data obtained in mice and humans. Joint efforts of several experts in the field resulted in formulation of guidelines for classification of macrophage phenotypes and polarization pathways [6, 26]. It was recommended to classify the different macrophage phenotypes based on the activation stimulus used and to avoid outdated terminology that could lead to confusion. It is currently unclear whether the results of* in vitro* experiments employing macrophage activation accurately reflect processes taking place* in vivo*, since macrophage activation may possibly be induced or modulated by macrophage isolation procedures. Moreover, the results obtained on experimental animals in many cases cannot be directly translated to humans, because the macrophage subtypes detected in different species (such as humans and mice) do not fully coincide. As macrophage activation is dependent on the expression of certain genes, studying changes of gene transcription in response to different stimuli will improve our understanding of this process. One of the important tools is the recently developed transcriptome analysis, which allows studying the complexity of macrophage activation variations [33]. A recent study has identified a network of transcriptional and epigenetic regulators that orchestrate the activation of proinflammatory macrophages [34]. The authors analyzed a variety of macrophage activation stimuli and proposed a model of human macrophage plasticity in inflammatory conditions defined by transcriptional regulation. Further study of the genetic mechanisms controlling macrophage activation may result in defining novel therapeutic targets for specific modulation of macrophage activation in pathological conditions.

## 4. The Role of Different Macrophage Types in Atherosclerosis

Atherosclerotic lesion site provides a specific microenvironment, enriched with activated cells, modified lipoproteins, and proinflammatory factors, as well as with dying and apoptotic cells. Correspondingly, the macrophage population of atherosclerotic plaques is heterogeneous [35]. The presence of relatively large numbers of proinflammatory macrophages (corresponding to M1 type) in atherosclerotic lesions is well known [28, 36]. However, alternatively activated macrophages have also been detected in the plaques [37]. Atherosclerotic plaque progression is associated with an increase of both macrophages populations, with cells expressing proinflammatory markers preferentially distributed in shoulder regions that are more susceptible to rupture and cells bearing markers of alternative activation located in the adventitia [38]. It has been demonstrated that anti-inflammatory, alternatively activated macrophages are present in more stable regions of plaques and are more resistant to foam cell formation [39]. Therefore, the pro- and anti-inflammatory macrophage subtypes may reflect the plaque progression/instability or regression correspondingly.

Identification of different types of macrophages in human tissues remains challenging because of the lack of specific and reliable markers. Immunohistochemical analysis of human aorta demonstrated the presence of proinflammatory macrophage marker TNF-*α* in atherosclerotic lesions as well as in grossly normal areas [40]. However, the quantity of TNF-*α* was increased in the lesion sites, which was also confirmed by quantitative PCR analysis of TNF-*α* expression. At the same time, atherosclerotic lesion areas also contained cells expressing CCL18, which are likely to be alternatively activated (M2-like) macrophages. More insight into macrophage polarization in proatherosclerotic conditions was gained by studying macrophage gene expression* in vitro*. Incubation of human monocyte-derived macrophages with multiply modified atherogenic LDL resulted in a significant increase of intracellular cholesterol accumulation associated with increased TNF-*α* and CCL18 expression [26].

Apart from the typical pro- and anti-inflammatory macrophages that can be classified into M1 and M2 types according to the old activation model, human atherosclerotic lesions contain specific macrophage phenotypes with pro- or antiatherogenic properties (Table 1). For instance, CD163-expressing macrophages could be found in haemorrhagic human plaques [41]. These cells are responsible for haemoglobin clearance and play a protective role in atherosclerotic lesions. Another atheroprotective macrophage subtype present in humans is Mhem. These cells also express CD163, as well as heme-dependent activating transcription factor 1 (ATF1), which induces expression of heme oxygenase 1 and liver X receptor- (LXR-) *β*. Mhem macrophages participate in haemoglobin clearance via erythrocyte phagocytosis and have increased cholesterol efflux due to expression of LXR-*β*-dependent genes LXR-*α* and ATP-binding cassette transporter 1 (ABCA1) [42]. These cells produce anti-inflammatory IL-10 and apolipoprotein E [43]. Recently described M4 macrophages can have proatherogenic properties and play a role in the formation of unstable plaques by producing MMP12 and promoting destabilization of the plaque fibrous cap [28].

## 5. Individual Difference in Macrophage Activation and Transcriptome Analysis

As mentioned above, human macrophages are characterized by great phenotypic diversity. Moreover, circulating monocytes can have unequal ability to polarize into different macrophage phenotypes, which can be relevant for atherosclerosis initiation and progression. It is important to evaluate susceptibility of circulating monocytes to pro- or anti-inflammatory polarization. For that purpose, monocytes were isolated from whole blood of healthy donors, apparently healthy subjects with predisposition to atherosclerosis, and patients with subclinical atherosclerosis evaluated by high-resolution ultrasonography of carotid arteries. Magnetic CD14-positive microbeads were used to obtain a pure monocyte population. Cells were stimulated with proinflammatory (IFN-*γ*) or anti-inflammatory (IL-4) factors [44]. In this simplified experimental model, the production of TNF-*α* and CCL18 was used as marker of pro- and anti-inflammatory activity, respectively, corresponding to M1 and M2 polarization of macrophages defined by the old paradigm. This approach revealed a remarkable individual difference in monocyte predisposition to activation [45]. This diversity did not, however, correlate with the presence or absence of subclinical atherosclerosis in the study subjects.

Transcriptome analysis is a powerful modern tool for studying monocyte/macrophage activation and function [23]. It provides a set of data on specific genes involved in different stages of macrophage activation. A detailed analysis of macrophage activation performed recently [33] explored changes in gene transcription induced by 28 different stimuli or their combinations. The study identified 49 sets of genes with similar transcriptional induction that become activated in macrophages in response to various stimuli and specific transcription factors that regulate them. More studies are needed, however, to reach an understanding of the complex mechanisms of macrophage activation* in vivo* [46]. It is likely that macrophage response to various stimuli in different individuals is largely influenced by genetic variation, especially in genomic regulatory elements that orchestrate the induced activation of macrophage genes. Such influence has recently been demonstrated, for instance, on F1 crosses of inbred mouse strains [47]. Memory of the past stimuli can also have a profound influence on monocyte ability for activation, as it has been demonstrated that some stimuli are not easily reversible and can influence the response of the immune system to subsequent stimulation [48].

A recent study has revealed an association of mitochondrial gene mutations with monocyte susceptibility to activation [49]. At least three heteroplasmic mutations of mtDNA, G14459A, A1555G, and G12315A, associated with atherosclerosis development in humans correlated with facilitated proinflammatory activation of circulating monocytes. Also, two homoplasmic mutations, A1811G and G9477A, tended to correlate with the degree of monocyte susceptibility to activation. On the other hand, G9477A mutation inversely correlated with the ability of monocytes to become activated. It is possible that mitochondrial dysfunction caused by mtDNA mutations activates autophagic clearance and contributed to the development of chronic inflammatory state, which also plays a role in the development of atherosclerosis. More studies are needed to evaluate the significance of mitochondrial genome for monocyte/macrophage system function.

## 6. Influence of Lipids on Macrophage Activation

LDL serves as the primary source of lipid accumulation in the arterial wall during atherosclerotic lesion development.* In vitro* studies have demonstrated that intracellular cholesterol accumulation is caused not by native but by atherogenic modified LDL. Unlike native LDL, modified LDL particles follow a different metabolic pathway, being internalized mostly through unregulated phagocytosis. Macrophages, with their well-developed phagocytic apparatus, are likely to play the key role in this process [28].

Both native and modified LDL were demonstrated to promote proinflammatory polarization of macrophages. A recent study on monocyte-derived macrophages has shown that incubation with LDL resulted in the increased expression of proinflammatory molecules TNF-*α* and IL-6 and decreased expression of CD206 and CD200R that are typical for anti-inflammatory (M2) macrophages [50]. Known forms of modified LDL also have a potent influence on macrophages promoting the formation of proinflammatory phenotype. Macrophages recognize modified LDL by means of TLRs and scavenger receptors. For instance, CD36, a scavenger receptor, can recognize oxidized LDL and associate with TLRs triggering proinflammatory signalling [51]. This favours macrophage polarization towards the proinflammatory phenotype. TLR activation is accompanied by the upregulation of protein kinases C and Syk, activation of NADPH oxidase 2 (gp91/Nox2), and increased ROS production [52]. As a result, macrophages increase the production of proinflammatory cytokines, including IL-1*β*, and chemokine (C-C motif) ligand 5 (CCL5). Moreover, oxidized LDL can induce inflammasome activation through CD36 signalling [53]. Exposure to oxidized LDL can also promote alternatively activated macrophages to shift their phenotype to a proinflammatory one through altered expression of pro- and anti-inflammatory genes [54].

It should be noted that the relationship between lipid accumulation and proinflammatory activation of macrophages is not straightforward. Lipidomic and transcriptomic study conducted in mice fed with normal or high cholesterol high fat diet demonstrated that macrophage-derived foam cells had a “deactivated” phenotype with reduced expression of proinflammatory factors [55]. Such anti-inflammatory response was attributed to cellular accumulation of desmosterol, one of the intermediates of cholesterol biosynthesis.

In mice, where a specific Mox type of macrophages has been described, oxidized phospholipids can induce both pro- and anti-inflammatory macrophage phenotypes to transform into Mox through activation of Nrf2, which promotes expression of a number of antioxidant genes [56]. Although Nrf2 signalling has some protective properties in atherosclerosis, its upregulation leads to inflammasome activation, which renders the Mox switch of macrophages proatherogenic [57]. Inflammasome activation in macrophages can result from phagocytosis of cholesterol crystals that can damage the lysosomal system [58]. Cholesteryl esters that are present in the plaque lipid core can stimulate macrophages and promote the inflammatory response and foam cell formation [52, 59]. The proinflammatory activity of different cholesteryl esters can be conveyed by different signalling pathways; for instance, 7-ketocholesteryl-9-carboxynonanoate was demonstrated to activate NF-*κ*B pathway [60] and cholesteryl linoleate-MAP kinase signalling [61]. Another proinflammatory class of cholesterol derivatives present in atherosclerotic plaques is oxysterol. In macrophages, oxysterol can induce the expression of proinflammatory monocyte chemoattractant-1 (MCP-1) [62] and scavenger receptor CD36 [63]. CD36 expression is also stimulated by oxidized cholesterol esters [64]. This scavenger receptor has an important role in atherogenesis, as its downregulation through stimulation of *α*M*β*2 integrins prevented the formation of proinflammatory macrophages and foam cells [65].

Phospholipase-mediated hydrolysis of lipoproteins resulting in the release of free phospholipids and fatty acids can occur in the acidic plaque microenvironment. These products greatly contribute to lipid accumulation in the arterial wall and plaque progression. It has been demonstrated that phospholipase A2-treated LDL increased the secretion of proinflammatory TNF-*α* and IL-6 by macrophages and stimulated foam cell formation [66]. The proinflammatory signalling of phospholipids and fatty acids is mediated by G-protein-coupled receptor G2A, which has an important role in the disease pathogenesis, as its deficiency results in advanced atherosclerosis and acquisition of proinflammatory M1 phenotype by macrophages [67]. Saturated fatty acids promote the proinflammatory phenotypic switch of macrophages through TLR-NF-*κ*B signalling [68].

Polyunsaturated fatty acids (PUFA) have well-known protective properties in atherosclerosis, which is partly explained by their anti-inflammatory effects on macrophages. Conjugated linoleic acid reduced the expression of proinflammatory genes such as NF-*κ*B, CCL2, MMP9, phospholipase 2, and cyclooxygenase 2 in macrophages through peroxisome proliferator-activated receptor *γ* (PPAR*γ*) and inhibited atherosclerosis progression in mice. PUFA can also counteract the proatherosclerotic effects of saturated fatty acids, such as palmitate-induced expression of lectin-like oxidized LDL receptor 1 (LOX1) and fatty acid-binding protein [69]. Eicosapentaenoic acid and dihydroascorbic acid (DHA) have protective effects in atherosclerosis by alleviating proinflammatory activity and improving functions of macrophages [70]. Nitro-fatty acids (NFA) can be formed by interaction of reactive nitrogen species with fatty acids during oxidative stress [71]. It has been demonstrated that NFA possess anti-inflammatory and atheroprotective properties mediated by Nrf2 and PPAR*γ* signalling [72]. Attenuation of atherosclerosis and plaque stabilization due to increased collagen deposition was observed in* Apoe* ^−/−^ mice treated with NFA [73].

High-density lipoprotein (HDL) has atheroprotective functions stimulating cholesterol efflux and catabolism [74]. Decreased relative levels of HDL versus LDL are observed in atherosclerotic patients. The protective effect of HDL is partly mediated by its anti-inflammatory activity: normalization of HDL serum levels in atherosclerotic mice led to a decrease of proinflammatory macrophage numbers in the lesions and to an increase of M2 macrophage markers CD163, Arg-1, and transcription factor FIZZ1 [75]. The expression of Arg-1 and FIZZ1 was dependent on STAT6 [76]. Another study has demonstrated that HDL inhibited the proinflammatory polarization of macrophages as assessed by such marker genes as TNF-*α*, IL-6, and CCL2, as well as surface markers, but did not stimulate the alternative activation of macrophages towards the anti-inflammatory phenotype [77]. Modulation of pro- and anti-inflammatory phenotypes of macrophages by lipids can be considered as a potential point of therapeutic intervention for treatment of atherosclerosis.

## 7. Foam Cell Formation

Intracellular accumulation of lipids is one of the early events in atherosclerosis development. Foam cell formation from macrophages is associated with downregulation of the expression of LDL receptor, which allows these cells to internalize apoB-containing lipoproteins. Modified LDL, which is internalized by alternative mechanisms, is the primary source of cholesterol accumulation in foam cells, as demonstrated by* in vitro* studies [78, 79]. Oxidation is the most studied atherogenic modification of LDL. It has been suggested that increased oxidative stress may account for the formation of atherogenic oxidized LDL and that the modified particle can trigger the development of the immune response and induce lipid accumulation in the arterial wall [80]. Studying of LDL composition of blood plasma of atherosclerotic patients revealed different types of LDL modification, including desialylation, glycation, acquisition of negative electric charge, and complex formation [81]. Complex formation renders modified LDL particles especially atherogenic. After penetration into the subendothelial layer of the arterial wall, modified LDL can associate with proteoglycan molecules, which increases its residence time and promotes lipid accumulation in the arterial wall cells. It is likely that a complex process of multiple LDL modification occurs in human bloodstream and in the arterial wall.

Modified LDL can be recognized by macrophages by means of scavenger receptors that play an important role in atherosclerosis development [82]. Scavenger receptors of macrophages include SR-A1, macrophage receptor with collagenous structure (MARCO, or SR-A2), CD36, SR-B1, LOX1, scavenger receptor expressed by endothelial cells 1 (SREC1), SR-PSOX, or CXCL16, recognizing phosphatidylserine and oxidized LDL [5].* In vitro* studies have shown that degradation of modified (acetylated or oxidized) LDL by macrophages is mediated mostly by SR-A1 and CD36 [78]. Deficiency of these receptors partly inhibited foam cell formation in* Apoe* ^−/−^ mice, suggesting that other mechanisms of LDL uptake exist in macrophages [83]. Large quantities of native LDL that can be observed in hyperlipidemic conditions of growing plaques can also contribute to foam cell formation being internalized via pinocytosis [84].

**Figure 2:**
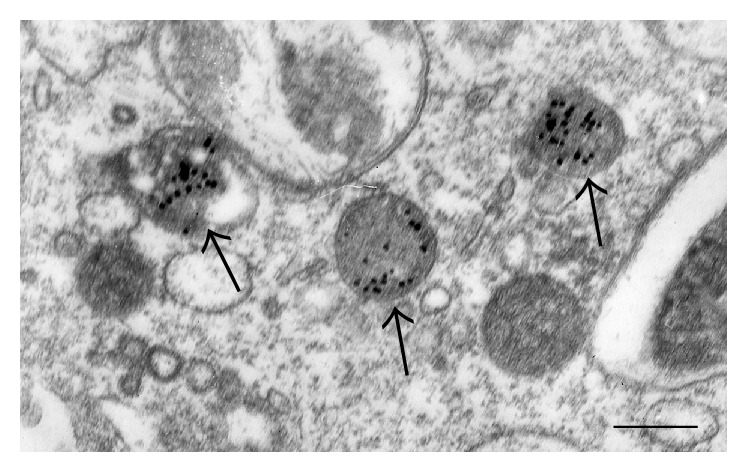
The presence of modified LDL, labelled with gold particles (arrows), in lysosomes of macrophages, visualized in an* in vitro* experiment. Transmission Electron Microscopy (TEM). Scale bar = 600 nm.

**Figure 3:**
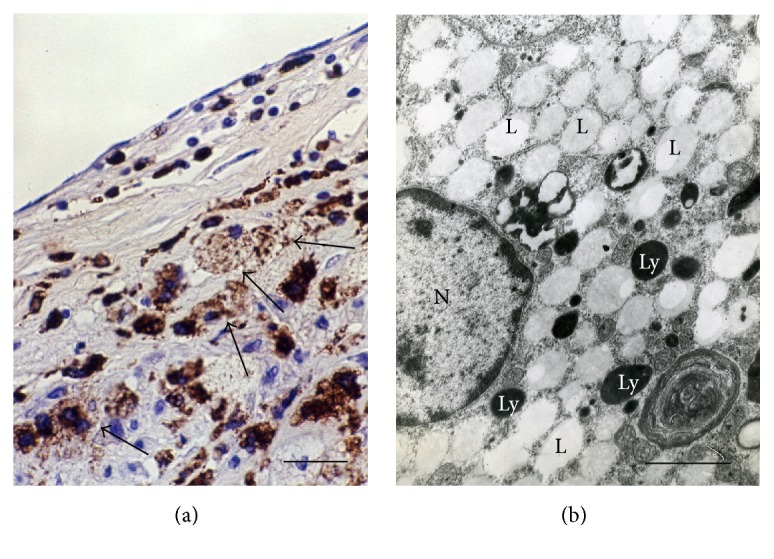
Foam cells of macrophage origin in an atherosclerotic lesion of the human aorta (a, b). (a) CD68+ cells (brown), some of which display a typical foam cell appearance (arrows). Immunohistochemistry; peroxidase-anti-peroxidase (PAP) technique; counterstain with Mayer's hematoxylin. (b) A large number of lipid inclusions (“lipid droplets”) (L) that fill practically all the cytoplasm in a foam cell in a human atherosclerotic plaque. Ly: lysosome; N: nucleus. TEM. Scale bars = 100 *μ*m (a) and 2 *μ*m (b).

Ultrastructural analysis of macrophages incubated with modified LDL* in vitro* experiments showed the accumulation of LDL in the lysosomes (Figures 2 and 3). Biochemical studies revealed that, after internalization, LDL particles are degraded in the lysosomal compartments to free cholesterol and fatty acids, and free cholesterol is trafficked to the endoplasmic reticulum (ER), where it is reesterified by acetyl-coenzyme A:cholesterol acetyltransferase 1 (ACAT1) [85, 86]. Excessive cholesterol uptake has deleterious effects on cells. Cholesterol accumulation in the ER membranes leads to its defective esterification by ACAT1 and further increased storage. ER stress associated with cholesterol storage in macrophages also contributes to the disease progression increasing apoptosis in progressing plaques [87]. Increased cell death and impaired clearance of dying cells result in the formation of necrotic core in advanced atherosclerotic plaques. Cholesterol-rich membrane microdomains facilitate proinflammatory TLR- and NF-*κ*B-mediated signalling [88].

It should be noted that LDL circulating in the blood of healthy individuals usually does not cause accumulation of lipids in cultured macrophages, whereas LDL of atherosclerotic patients is in most cases a potent inducer of cellular lipidosis. Thus, LDL of atherosclerotic patients, unlike LDL of healthy individuals, is atherogenic. When added to primary culture of human monocyte-derived macrophages, atherogenic LDL isolated from the blood of atherosclerotic patients induces upregulation of proinflammatory cytokine TNF-*α* and anti-inflammatory chemokine CCL18 at the transcription level (Table 2). At the same time, nonatherogenic (native) LDL from healthy individuals had no effect on gene expression when added to cultured macrophages (Table 2). Therefore, multiply modified atherogenic LDL causes pro- and anti-inflammatory macrophage activation. This is a very important observation considering the significant role of the innate immunity and chronic inflammation in the occurrence and development of atherosclerotic lesions. The findings are in good agreement with the results of* in situ* studies that have demonstrated upregulation of the expression of pro- and anti-inflammatory cytokines in atherosclerosis [35, 89].

To assess the impact of modified LDL-induced cholesterol accumulation on gene expression in macrophages, transcriptome study of macrophages incubated with oxidized, acetylated, and desialylated LDL was performed. Naturally, the addition of modified LDL caused changes in the activity of hundreds of macrophage genes. It is important to identify the genes that are associated with lipid accumulation. Incubation with modified LDL altered the activity of forty genes encoding molecules with known functions (Table 3). It should be noted that most of these genes (26 of 40) may be related to innate immunity function. This observation suggests that LDL-induced cholesterol accumulation in macrophages triggers an immune response. Further research should explain the link between the intracellular lipid accumulation and chronic inflammation in atherosclerotic lesions.

Increased lipid efflux could be a powerful therapeutic option for treatment of atherosclerosis. Several proteins facilitate lipid efflux in macrophages, including ABCA1 and ABCG1 [90]. ABCA1 and ABCG1 mediate lipid efflux to HDL particles and are upregulated in response to increased cellular cholesterol levels sensed by liver X receptors (LXRs). Their activation has also anti-inflammatory effects [91]. It was demonstrated that LXR activation in murine macrophages lacking ABCA1/G1 had a strong antiatherosclerotic effect, decreasing lesion area and complexity through reduction of inflammation [92]. The therapeutic option of activating LXRs for treatment of atherosclerosis is currently being explored. Another mechanism of cholesterol clearance from cells is lipophagy, which is a special type of autophagy [93]. Studies on atherosclerosis mouse model demonstrated the protective role of autophagy through regulation of inflammation [94] and cell death [95] in atherosclerotic plaques.

## 8. Macrophage-Based Tests for Diagnostics and Search of Antiatherosclerotic Substances

Given the crucial role that macrophages play in the parthenogenesis of atherosclerosis, it is important to establish reliable monocyte/macrophage-based models that can be used for studying molecular mechanisms of the disease pathogenesis as well as for screening of potential antiatherosclerotic substances. Recently, a monocyte/macrophage-based assay was developed to evaluate the changes in patient's monocyte response to pro- and anti-inflammatory stimuli, which would reveal the possible bias of the macrophages polarization towards M1 or M2 phenotype. A pure population of monocytes/macrophages was obtained using magnetic separation method [96]. Isolated cells were stimulated with proinflammatory LPS and IFN-*γ* or with anti-inflammatory IL-4 [97]. Macrophage polarization was assessed by measuring the production of pro- and anti-inflammatory cytokines by ELISA. Proinflammatory activity of macrophages was assessed by the levels of secreted TNF*α* and IL-1*β*, and anti-inflammatory activity was assessed by the levels of CCL18 and IL-1Ra. Inflammasome activation can also be assessed in this system by measuring the IL-1*β* expression at mRNA level and comparing the results with the amount of mature IL-1*β* detected by ELISA or by measurement of active caspase-1, TNF-*α*, and IL-8 [98]. Characterization of macrophages can be performed by the analysis of a panel of markers, including MMR, CD163, TGF-RII, CSFR1, TNFRI, CD16, CD32, CD64, and stabilin-1, as well as expression of TLR1, TLR2, and TLR4 at mRNA level and on the cell surface.

Monocyte/macrophage-based method was used to analyze activation of monocytes isolated from blood of healthy subjects (*n* = 19), atherosclerosis patients (*n* = 22), and breast cancer patients (*n* = 18). It was demonstrated that the production of proinflammatory TNF-*α* was significantly lower in atherosclerotic patients and significantly higher in cancer patients in comparison to healthy subjects, whereas the production of anti-inflammatory CCL18 was decreased in both atherosclerosis and cancer patients [40]. Comparison of subjects with predisposition to atherosclerosis (*n* = 21, mean age 63 ± 9 years), subjects with subclinical atherosclerosis (*n* = 21, mean age 62 ± 7 years), and healthy subjects (*n* = 21, mean age 60 ± 9 years), as estimated by the age-adjusted carotid intima media thickness (CIMT) value, revealed the dramatic individual differences between the analyzed subjects that may reflect the individuals' predisposition to immunopathology [40]. Macrophages from subjects with subclinical atherosclerosis were characterized by especially low degree of polarization towards pro- and anti-inflammatory phenotypes.

Macrophage-based model could also be successfully used for evaluation of potential antiatherosclerotic substances. The ability of botanicals with known anti-inflammatory properties to modulate the activation of macrophages was evaluated using IFN-*γ* and IL-4 stimulation. Cultured human macrophages were incubated with extracts of hawthorn flowers (*Crataegus *sp.), elderberry (*Sambucus nigra*), calendula (*Calendula officinalis*), St. John's wort (*Hypericum perforatum*), and violet (*Viola *sp.), and the levels of TNF-*α* and CCL18 were measured after 6 days. Extracts of hawthorn and St. John's wort significantly inhibited both TNF-*α* and CCL18 production indicative of macrophage depolarization [40]. This interesting immunomodulatory effect should be explored in more detail to reveal its possible therapeutic significance.

## 9. Conclusion

Macrophages play a central role in the pathogenesis of atherosclerosis. They actively participate in LDL uptake and lipid accumulation in the arterial wall becoming foam cells. Macrophage population is heterogeneous and consists of several subtypes of cells that differ by their functions and gene expression profiles. Proinflammatory macrophages are implicated in plaque initiation and progression, while anti-inflammatory macrophages participate in plaque stabilization. Monocytes/macrophages isolated from the blood of healthy subjects and atherosclerotic patients can accumulate lipids upon incubation with atherogenic LDL and can be used to create cell-based models for evaluation of potential antiatherosclerotic substances. Interestingly, monocytes/macrophages isolated from blood demonstrated a significant interindividual variability, which could possibly be explained by varying gene regulation and previous history of immune cells activation. Given the importance and variety of macrophage functions in atherosclerosis, these cells are considered an attractive therapeutic target. Future studies should focus on further investigation of the roles of different types of macrophages in atherosclerosis progression and on development of macrophage-targeting therapies.

## Figures and Tables

**Table 1:** Macrophage phenotypes detected in humans and mice and their role in atherosclerosis  *(adapted with modifications from [26], with permission from Elsevier)*.

Phenotype	Induction	Markers	Secreted molecules	Functions	Role in atherosclerosis
M1 (human, mouse)	IFN-*γ*, TNF-*α*, LPS, and other TRL-mediated stimuli	IL-1*β*, IL-6, IL-12, IL-23, TNF-*α*, CXCL9, CXCL10, and CXCL11	IL-6, IL-10 (low), IL-12 (high), IL-23, TNF-*α*, iNOS, and ROS	Th1 response, antitumor	Plaque progression, maintaining inflammatory response
M2a (human, mouse)	IL-4, IL-13	Human: MR, IL1RN Mouse: Arg-1, FIZZ1, and Ym1/2	IL-10, TGF-*β*, CCL17, and CCL22	Tissue repair and remodelling	
M2b (human, mouse)	IL-1*β*, LPS	IL-10 (high), IL-12 (low)	IL-6, IL-10 (high), IL-12 (low), and TNF-*α*	Immune regulatory functions	Enriched in regressing plaques in humans and mice
M2c (human, mouse)	IL-10, TGF-*β*, and glucocorticoids	Human: MR Mouse: Arg-1	IL-10, TGF-*β*, and PTX3	Phagocytosis, apoptotic cell clearance	
M2d (mouse)	TLR + A_2_R ligands	IL-12 (low), TNF-*α* (low)	IL-10, VEGF, and iNOS	Angiogenesis	Present in murine plaques
M4 (human)	CXCL4	MR, MMP7, and S100A8	IL-6, TNF-*α*, and MMP12	Weak phagocytosis	Minimal foam cell formation, potentially proatherogenic
Mox	Oxidized LDL	HMOX-1, Nrf2, Srxn1, and Txnrd1	IL-1*β*, IL-10	Weak phagocytosis	Proatherogenic properties in mice
HA-mac (human)	Haemoglobin/ haptoglobin	CD163 (high), HLA-DR (low)	HMOX-1	Haemoglobin clearance	Atheroprotective
M (Hb) (human)	Haemoglobin/ haptoglobin	MR, CD163	ABCA1, ABCG1, and LXR*α*		Cholesterol efflux, atheroprotective
Mhem (human, mouse)	Heme	ATF1, CD163	LXR*β*	Erythrocyte phagocytosis	Atheroprotective

**Table 2:** Effect of LDL on cytokine gene expression.

	Native LDL	Atherogenic LDL
TNF-*α*	1.0 ± 0.3 (1.11)	2.0 ± 0.5 (2.1) *P* = 0.05
CCL18	1.1 ± 0.5 (1.0)	4.4 ± 0.9 (2.8) *P* = 0.03

Monocytes were isolated from whole blood of healthy donors by density gradient followed by selection of CD14^+^ cells by magnetic separation. Cells were cultured for 7 days. Native or atherogenic LDL was added at a concentration of 100 *µ*g/mL and the cells were incubated for 24 hours. RNA was isolated and gene expression was measured by RT-PCR technique. The table shows the relative expression of the genes. As 1, the control gene expression (without LDL) was taken. Values in parentheses are standard deviations.

**Table 3:** List of macrophage genes whose activity changes in the accumulation of intracellular cholesterol.

Gene	Molecule	Functions
FCGBP	Fc fragment of IgG binding protein	Immune response
S100A8	S100 calcium binding protein A8	Immune response, migration, cell body formation
ITLN1	Intelectin 1 (galactofuranose binding)	Pathogen metabolism
NCOR2	Nuclear receptor corepressor 2	Immune response
TPPP3	Tubulin polymerization-promoting protein family member 3	Cell body formation
AKR1C1	Aldo-keto reductase family 1, member C1	Immune response
FAM65A	Family with sequence similarity 65, member A	Cell body formation
HECTD2	HECT domain containing E3 ubiquitin protein ligase 2	Metabolism
RD3	Retinal degeneration 3	Nerve features
TNFSF18	Tumor necrosis factor (ligand) superfamily, member 18	Immune response, migration
NEURL3	Neuralized E3 ubiquitin protein ligase 3	Metabolism
CD209	CD209 molecule	Immune response, migration, dendritic cell features
STRIP2	Striatin interacting protein 2	Cell body formation
CCL4L2	Chemokine (C-C motif) ligand 4-like 2	Migration
TJP2	Tight junction protein 2	Migration
SPON2	Spondin 2, extracellular matrix protein	Migration
L1CAM	L1 cell adhesion molecule	Migration
ARHGEF16	Rho guanine nucleotide exchange factor (GEF) 16	Migration
NES	Nestin	Cell body formation, nerve features
F3	Coagulation factor III (thromboplastin, tissue factor)	Migration
GALNT5	Polypeptide N-acetylgalactosaminyltransferase 5	Metabolism
MT1E	Metallothionein 1E	Metabolism
COQ2	Coenzyme Q2 4-hydroxybenzoate polyprenyltransferase	Metabolism
TRIM54	Tripartite motif containing 54	Cell body formation
ANKRD63	Ankyrin repeat domain 63	Cell body formation
CCL24	Chemokine (C-C motif) ligand 24	Immune response, migration
HIVEP3	Human immunodeficiency virus type I enhancer binding protein 3	Immune response
NETO2	Neuropilin (NRP) and tolloid- (TLL-) like 2	Nerve features
CCL4	Chemokine (C-C motif) ligand 4	Immune response, migration
ACPP	Acid phosphatase, prostate	Metabolism
STARD4	StAR-related lipid transfer (START) domain containing 4	Metabolism
RANBP10	RAN binding protein 10	Cell body formation
ROBO2	Roundabout guidance receptor 2	Migration, nerve features
CHL1	Cell adhesion molecule L1-like	Migration, nerve features
RARA	Retinoic acid receptor, alpha	Negative regulation of interferon-gamma production; positive regulation of interleukin-4 production, immune response
SLC16A9	Solute carrier family 16, member 9	Metabolism
HTR2A	5-Hydroxytryptamine (serotonin) receptor 2A, G-protein-coupled	Nerve features
BCAR1	Breast cancer antiestrogen resistance 1	Migration
OR6K3	Olfactory receptor, family 6, subfamily K, member 3	Nerve features
CYP7B1	Cytochrome P450, family 7, subfamily B, polypeptide 1	Metabolism
